# Respective Contribution of Chronic Conditions to Disability in France: Results from the National Disability-Health Survey

**DOI:** 10.1371/journal.pone.0044994

**Published:** 2012-09-14

**Authors:** Clémence Palazzo, Jean-François Ravaud, Ludovic Trinquart, Marie Dalichampt, Philippe Ravaud, Serge Poiraudeau

**Affiliations:** 1 U738, INSERM, Paris, France; 2 Centre d'Épidémiologie Clinique, Hôpital Hôtel Dieu AP-HP, Paris, France; 3 Université Paris Descartes, PRES, Paris, France; 4 Service de rééducation et réadaptation de l'appareil locomoteur et des pathologies du rachis, Hôpital Cochin AP-HP, Paris, France; 5 Institut fédératif de recherche sur le handicap, INSERM, Paris, France; 6 U988, INSERM, Villejuif, France; 7 UMR 8211, CNRS, Villejuif, France; UCSD School of Medicine, United States of America

## Abstract

**Background:**

Representative national data on disability are becoming increasingly important in helping policymakers decide on public health strategies. We assessed the respective contribution of chronic health conditions to disability for three age groups (18–40, 40–65, and >65 years old) using data from the 2008–2009 Disability-Health Survey in France.

**Methods:**

Data on 12 chronic conditions and on disability for 24,682 adults living in households were extracted from the Disability-Health Survey results. A weighting factor was applied to obtain representative estimates for the French population. Disability was defined as at least one restriction in activities of daily living (ADL), severe disability as the inability to perform at least one ADL alone, and self-reported disability as a general feeling of being disabled. To account for co-morbidities, we assessed the contribution of each chronic disorder to disability by using the average attributable fraction (AAF).

**Findings:**

We estimated that 38.8 million people in France (81.7% [95% CI 80.9;82.6]) had a chronic condition: 14.3% (14.0;14.6) considered themselves disabled, 4.6% (4.4;4.9) were restricted in ADL and 1.7% (1.5;1.8) were severely disabled. Musculoskeletal and sensorial impairments contributed the most to self-reported disability (AAF 15.4% and 12.3%). Neurological and musculoskeletal diseases had the largest impact on disability (AAF 17.4% and 16.4%, respectively). Neurological disorders contributed the most to severe disability (AAF 31.0%). Psychiatric diseases contributed the most to disability categories for patients 18–40 years old (AAFs 23.8%–40.3%). Cardiovascular conditions were also among the top four contributors to disability categories (AAFs 8.5%–11.1%).

**Conclusions:**

Neurological, musculoskeletal, and cardiovascular chronic disorders mainly contribute to disability in France. Psychiatric impairments have a heavy burden for people 18–40 years old. These findings should help policymakers define priorities for health-service delivery in France and perhaps other developed countries.

## Introduction

Disability is fast becoming a concern because of its increasing prevalence owing to the aging of the population, the increased risk of disability in older people, and the global increase in chronic conditions [1]. According to the 2004 Global Burden of Disease estimates [2], chronic non-communicable diseases contributed to 68% of 751 million years lived with disability worldwide and were largely depression, sensorial impairments and osteoarthritis in elderly people.

Preferences in public health priorities differ when the target is mortality or disability. Studies of the contribution of different diseases to mortality have been numerous, but those investigating the contribution of disability are lacking. Several authors compared the contribution of different disorders to disability [3], [4], [5], [6] to help policymakers decide on intervention and preventive strategies. However, the studies vary in terms of groups studied, diseases and approaches used to define and measure disability. Moreover, these data often concern co-morbid situations, so a reliable estimate of the respective weight of diseases in global disability is difficult. This situation is particularly true for studies in developed countries and involving elderly people.

Use of the average attributable fraction (AAF) [7] has been recently suggested to solve the problem of the respective weight of diseases to global disability [8]. Briefly, the AAF is the expected proportion of cases of disability preventable by the additional elimination of the condition of interest after a random collection of other disorders has been eliminated [7].

In 2008–2009, the national representative Disability-Health Survey was carried out to describe disabilities in the population in France. We used the AAF to analyze data from this survey to assess the current contribution of chronic conditions to disability.

## Methods

### Ethics

This study was planned as a research project. It was performed in collaboration with the French National Institute of Statistics. This study was declared of public interest by the CNIS (Conseil National d'Information Statistique) and was approved by the CNIL (Commission Nationale de l'Informatique et des Libertés, French law no. 78-17). According to the French law, written informed consent was not required for this type of study.

### Disability-Health Survey design

**Figure 1:**
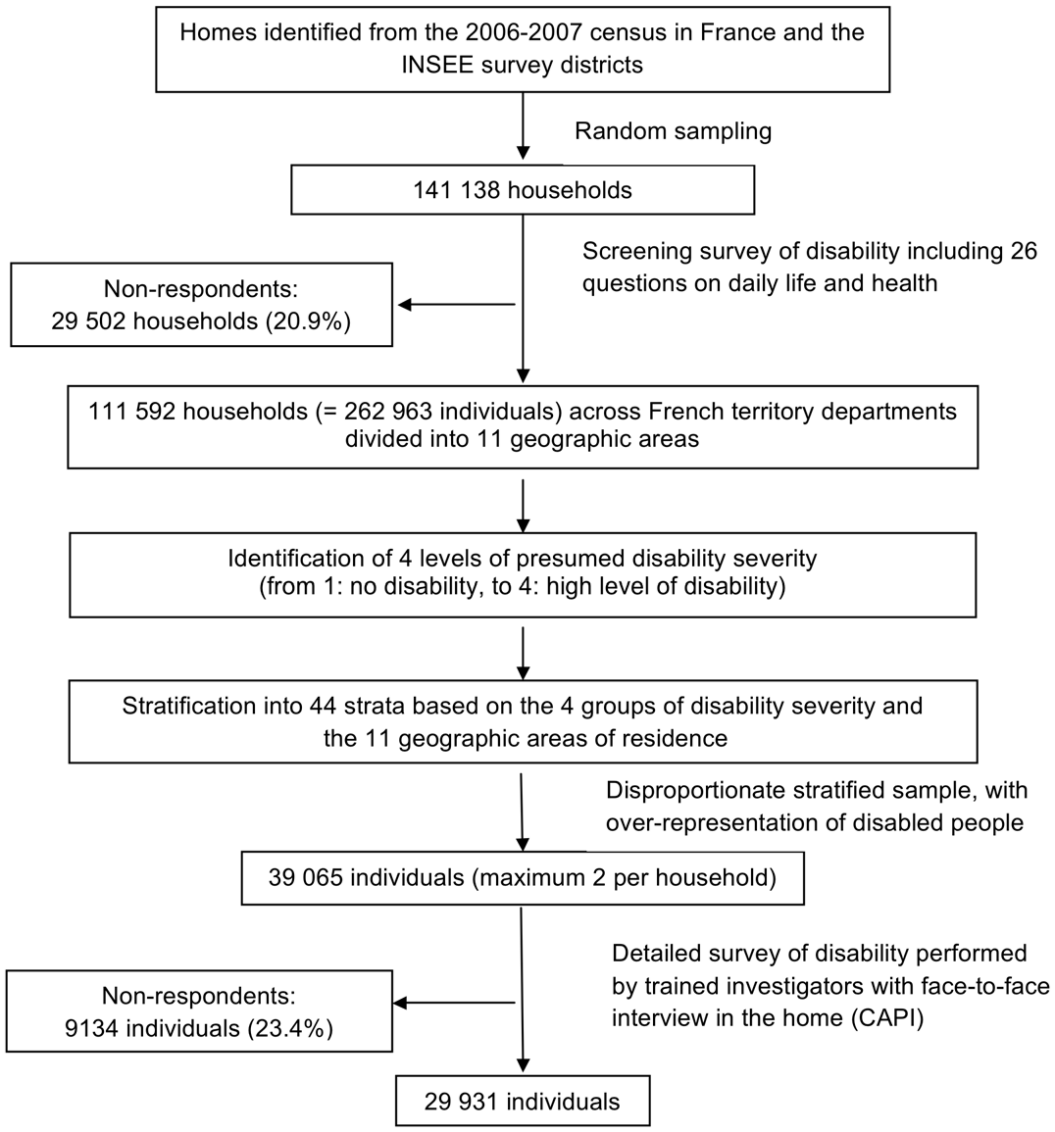
Design of the representative national “Disability-Health” survey. INSEE = French National Institute of Statistics and Economic Studies.

The Disability-Health Survey (available at http://www.sante.gouv.fr/handicap-sante.html) was a national cross-sectional survey with a two-stage design (Figure 1) aiming to describe disability and handicap in France. It was developed by the French National Institute of Statistics and Economic Studies (INSEE) and the French Head Office of Research, Studies, Evaluation and Statistics of the Social Affairs Ministry.

First, a preliminary filter-survey was performed to identify and stratify disabilities. Briefly, a questionnaire was sent to 141 138 representative households from the 2006 census in France and the INSEE survey districts. The response rate was 79.1%. The questionnaire has 26 sections on health, activity limitations, help, administrative recognition of disability, and personal perception of the situation. According to their answers, people were classified into four levels of presumed disability severity, from 1 (no disability) to 4 (high level of disability). The survey also involved intensive sampling in several geographic areas to obtain representative data in these areas. Stratification into 44 strata was based on the four presumed disability levels and the 11 geographic areas of residence.

For sample selection, randomisation involved a high sampling rate for the most severely disabled group and a low sampling rate for people without daily living restrictions (the largest group). Each of the resulting groups was allocated a specific sampling coefficient that increased with the probability or severity of the presumed handicap. The sampling rate was also higher for people living in the geographic areas that were more intensively sampled. Data were collected from March to July 2008, including 39 065 individuals across the territory departments in France. Trained investigators used the computer-assisted interview (CAPI) format to collect data from people in their homes. A household member or a proxy could answer for identified survey respondents not able to answer alone. The response rate was 76.6%, corresponding to 29 931 subjects (24 682 adults older than 18 years) with complete data.

Each respondent was assigned a weight reflecting the probability of being investigated (depending on presumed disability severity and geographic area of residence) and answering the questionnaire, which allowed for estimating representative results at a national level.

### Measurement of disability

We considered three situations of disability on the basis of subjects' self-reports.

We defined overall disability and severe disability according to Katz' activities of daily living (ADL) score [9], which includes six items: bathing, dressing, toileting, transferring (moving from bed and chair), preparing a meal and self-feeding. Respondents were asked about the degree of difficulty in performing these activities on a scale from 0 (no difficulty) to 4 (inability to do it alone). Disability was defined as any restriction in performing ADL with or without technical aid [10] and severe disability as the inability to perform at least one ADL alone [11]. Self-reported disability was considered a positive answer to the question “Do you consider that you have a disability?”

### Assessment of chronic conditions and socio-demographic characteristics

Chronic diseases were self-reported. Interviewers presented a checklist of 52 disorders classified in 12 groups according to the 10^th^ International Classification of Diseases criteria [12]: cardiovascular, musculoskeletal, neurological, psychiatric, respiratory, dermatological, endocrine, urological, digestive, and sensorial diseases; cancer; and sequelae of injury (Table 1).

**Table 1:** Chronic conditions and the included diseases evaluated in estimating the contribution of diseases to disability in the population in France.

Chronic conditions	Including diseases
**Cancer**	cancer (including lymphoid, haematopoietic and related tissue)
**Cardiovascular**	myocardial infarction, angina, stroke, heart failure, lower limb arterial occlusive disease, venous insufficiency and high blood pressure
**Dermatological**	psoriasis, eczema and slough
**Digestive**	ulcer, cirrhosis (and other liver diseases) and food allergies
**Endocrine**	diabetes mellitus, disorders of the thyroid gland and obesity (body mass index ≥30 kg/m^2^)^a^
**Musculoskeletal**	back pain, neck pain, scoliosis, rheumatoid arthritis, other inflammatory arthritis, knee and hip osteoarthritis, other type of osteoarthritis and osteoporosis
**Neurological**	headache, epilepsy, dementia, Parkinson's disease, multiple sclerosis and other unspecified neurological problems
**Psychiatric**	depression, anxiety, autism, schizophrenia, trisomy 21 and other unspecified psychiatric impairments
**Respiratory**	asthma, chronic bronchitis and hay fever
**Sensorial**	eyesight problems ^b^ and hearing loss^c^
**Sequelae of injury**	sequelae of injury
**Urological**	urinary incontinence, infection of the urinary tract, lithiasis and prostate adenoma

This classification followed the 10^th^ International Classification of Diseases [12]; ^a^ Calculated from reported height and weight as weight/height^2^; ^b^ Eyesight problems included cataract, strabismus and glaucoma reported in the checklist of the questionnaire and a positive answer to the question: “Do you have any eyesight problems?”; ^c^ Hearing loss included a positive answer to the questions: “Are you wearing a hearing aid?” or “Do you have any hearing problems?”

The diagnosis of condition had to be from a physician, except for symptoms such as back pain, neck pain and headache. We also assumed disorders not treated during the previous year and stroke without any sequelae as “no disease.”

The survey collected data on sex, age, marital status, educational attainment, working status, occupation class, place of residence (urban/rural), and living situation (alone or not).

### Statistical analysis

The final weighting factors combined design weights and non-response weights. Design weights were the inverse of the sampling fraction, depending on presumed disability severity and geographic area of residence. Probability of non-response was estimated by logistic regression, with age, gender, type of household, marital status, and nine questions about health and disability used as independent variables. Finally, calibration was based on geographic area of residence, age and gender.

For the descriptive analysis, we reported the prevalence of diseases, summarized socio-demographic characteristics and described disabilities by frequencies, means and their 95% confidence intervals (95% CIs) corrected with the sampling weight.

To assess the respective contribution of chronic diseases to disability, we used three steps. First, we calculated the gross attributable fraction (AF) of each chronic disorder to disability, defined as the fraction of the overall rate of disability that could be avoided if that disease was eliminated in the population [13]. AFs were calculated using Levin's formula [13]:
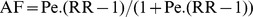
Pe is the prevalence of the disease and RR the relative risk estimate to be disabled when having the disease. The 95% confidence intervals were calculated with Fleiss' formula [14]. The main limitation of AF is that it measures not the single contribution of the disease of interest. Considering an example from our dataset, an AF of 20% for musculoskeletal impairment means that for 20% of people reporting disability, the disability can be globally attributed to musculoskeletal disorders; that is to musculoskeletal disorders alone, but also to musculoskeletal disorders associated with other chronic disease(s), such as neurological or psychiatric conditions. Consequently, AF can overestimate the potential impact of musculoskeletal disorders [8], and the sum of the AFs of all chronic conditions can add up to more than 100%, which can produce some confusion when interpreting the results. We grouped diseases with a low AF (arbitrarily ≤15%; “other diseases”) for the following calculations.

Next, we calculated the AAF [7], considered to be a relevant methodology for use in co-morbid situations, and defined as the expected proportion of disability preventable by the additional elimination of the chronic condition of interest, after adjustment for a random collection of other disorders [7]. Briefly, the AAF is based on the idea of partitioning disability into a set of risk factors, including chronic disorders. For the previous example, an AAF of 20% for musculoskeletal disorders means that for 20% of people reporting disability, the disability can be attributed only to musculoskeletal diseases. Contrary to AF, the AAF of a chronic disorder reflects its single contribution to disability, and the sum of the AAFs of all chronic conditions should not add up to more than 100%. The method of calculating the AAF is explained in detail with an example in Appendix S1. To our knowledge, no method exists for calculating AAF confidence intervals.

Finally, AAFs were multiplied by the total number of disabled subjects to estimate the expected number of disabled subjects in whom disability would be prevented by eliminating each chronic disorder.

Because prevalence rates of diseases and frequencies of disability vary by age, we analyzed these categories and AAFs in the overall population by three age groups: 18–40, 40–65, and >65 years. We adjusted for potential confounders identified a priori from the literature: sex [15], marital status [16], living situation (alone or not) [17], educational level [18] and place of residence (rural area, urban area <200 000 people and urban area ≥200 000 people) [19].

Statistical analyses involved use of SAS 9.2 (SAS Inst, Cary, NC). Sampling weights were taken into account with specific SAS procedures for handling complex sample designs [20]. AAFs were computed with the macro developed by Rückinger et al [8] that we modified to take into account sample design and adjustment for variables.

## Results

### Prevalence rates and socio-demographic characteristics of people with chronic conditions in France

The prevalence rates and socio-demographic characteristics of survey subjects reporting chronic diseases are in Table 2 (in detail in Table S1).

**Table 2:** Prevalence rates and socio-demographic characteristics of patients reporting chronic conditions in the Disability-Health Survey and population estimates.

Chronic conditions	No. of survey subjects (n = 22 332)^a^	Prevalence in the population with chronic diseases (%) (95% CI)	Prevalence in the overall adult population (%) (95% CI)	Estimated no.^c^ of people in the population with chronic diseases (*N = 38 837 743*)^b^	Mean age (years) (95% CI)	Frequency of men (%) (95% CI)	Mean no. of chronic conditions (95% CI)
**Cancer**	922	2.4	1.9	923 096	63.3	47.6	3.7
		(2.1;2.6)	(1.7;2.2)		(61.6;64.9)	(42.0;53.1)	(3.5;3.9)
**Cardiovascular**	8385	26.0	21.2	10 095 211	63.3	42.0	3.4
		(25.2;26.8)	(20.6;21.9)		(62.8;69.9)	(40.3;43.8)	(3.4;3.5)
**Dermatological**	1780	7.6	6.2	2 957 850	45.9	40.7	3.4
		(7.0;8.2)	(5.8;6.7)		(44.7;47.2)	(36.8;44.5)	(3.3;3.5)
**Digestive**	1966	5.6	4.6	2 179 675	54.4	41.6	4.2
		(5.2;6.0)	(4.2;4.9)		(53.0;67.0)	(37.7;45.5)	(4.0;4.3)
**Endocrine**	7071	23.0	18.8	8 932 739	56.1	41.0	3.2
		(22.2;23.8)	(18.1;19.5)		(55.5;56.7)	(39.0;43.0)	(3.1;3.2)
**Musculoskeletal**	10 648	35.2	28.7	13 656 107	57.5	38.8	3.3
		(34.2;36.1)	(27.9;29.5)		(57.0;58.0)	(37.2;40.4)	(3.2;3.3)
**Neurological**	4144	12.4	10.1	4 796 960	49.0	33.1	3.5
		(11.7;13.0)	(9.6;10.6)		(48.1;49.9)	(30.5;35.8)	(3.4;3.6)
**Psychiatric**	3371	7.7	6.3	2 983 786	54.6	33.5	4.0
		(7.2;8.1)	(5.9;6.7)		(53.6;55.6)	(30.6;36.5)	(3.9;4.1)
**Respiratory**	3668	14.0	11.5	5 443 578	49.5	43.1	3.3
		(13.3;14.7)	(10.9;12.1)		(48.6;50.4)	(40.4;45.9)	(3.2;3.4)
**Sensorial**	18 447	80.1	65.4	31 098 284	53.7	44.1	2.4
		(79.2;81.0)	(64.5;66.4)		(53.3;54.1)	(42.9;45.3)	(2.4;2.4)
**Sequelae of injury**	1511	4.3	3.5	1 683 703	51.7	59.5	3.7
		(4.0;4.7)	(3.2;3.9)		(50.1;53.3)	(55.2;63.7)	(3.6;3.9)
**Urological**	2147	5.9	4.8	2 289 618	60.5	36.6	4.1
		(5.5;6.3)	(4.5;5.2)		(59.0;62.0)	(33.0;40.2)	(4.0;4.2)

95% CI = 95% confidence interval; ^a^ n = number of people living in a household and reporting at least 1 chronic condition; ^b^ *N* = estimated number of people in the French adult population living in a household and reporting at least 1 chronic condition; ^c^ Numbers reflect non-rounding used for calculations.

The mean age of the population was 48.4 years old (95% CI 48.1;48.7). In the overall household population (47 524 123 people): 37.7% (36.7;38.7) were 18 to 40 years old, 42.8% (41.8;43.8) were 40 to 65 years old and 19.5% (18.8;20.1) were >65 years old. In the overall household population, an estimated 38.8 million people 81.7% (80.9;82.6) had chronic conditions. The most frequently reported conditions were sensorial and musculoskeletal impairments. These impairments were reported by 80.1% (79.2;81.0) and 35.2% (34.2;36.1), respectively, of the population with chronic conditions and by 65.4% (64.5;66.4) and 28.7% (27.9;29.5), respectively, of the overall adult population.

People with chronic conditions were older than those in the overall adult population (mean age 51.4 (51.0;51.8) vs. 48.4 (48.1;48.7) years). People >65 years old represented 23.2% (22.5;23.9) of people reporting chronic diseases and 19.5% (18.9;20.1) of the overall adult population. This proportion was high for people reporting cancer (46.5% (41.1;51.9)), cardiovascular diseases (46.8% (45.2;48.5)) and urological impairments (47.6% (44.2;51.0)). The mean number of chronic diseases was high for urological (4.1 (4.0;4.2)) and digestive disorders (4.2 (4.0;4.3)). Chronic conditions were less frequent for men, except for sequelae of injury. Almost half of the estimated 3 million people with psychiatric disorders were single (44.9% (41.9;47.9)). People with psychiatric and neurological diseases had high unemployment rates (6.9% (5.4;8.3) and 7.0% (5.6;8.5), respectively).

### Frequencies of chronic conditions with disability in the overall adult population

In total, 12.0% (11.8;12.3) of people (approximately 5.7 million people) considered that they had a disability, with sensorial and musculoskeletal disorders the main chronic diseases (82.8% (81.7;83.9) and 56.3% (54.9;57.7), respectively) (Table 3). In all, 3.9% (3.7;4.1) (approximately 1.8 million people) were restricted in at least one ADL, particularly bathing and dressing, with sensorial and musculoskeletal conditions the most frequently reported diseases (85.2% (83.3;87.2) and 63.3% (60.7;65.9)). Finally, 1.4% (1.3;1.5) (approximately 0.67 million people) reported severe disability, with sensorial and cardiovascular disorders the most frequently reported diseases (82.6% (79.0;86.3) and 53.3% (48.9;57.6)).

**Table 3:** Estimated frequency of each chronic condition in different situations of disability for people in France based on responses to the Disability-Health Survey.

	Self-reported disability^a^ (%) (95% CI)	Disability^b^ (%) (95% CI)	Severe disability^c^ (%) (95% CI)
**Population with chronic diseases**	**14.3 (14.0;14.6)**	**4.6 (4.4;4.9)**	**1.7 (1.5;1.8)**
**Total population** d	**12.0 (11.8;12.3)**	**3.9 (3.7;4.1)**	**1.4 (1.3;1.5)**
**Cancer**	4.9 (4.3;5.5)	5.2 (3.8;6.6)	5.5 (3.2;7.8)
**Cardiovascular**	43.8 (42.4;45.2)	53.2 (50.6;55.8)	53.3 (48.9;57.6)
**Dermatological**	8.8 (8.0;9.5)	9.7 (8.4;11.1)	8.2 (6.7;9.7)
**Digestive**	10.5 (9.6;11.3)	11.5 (9.9;13.0)	10.4 (7.6;13.1)
**Endocrine**	34.4 (33.1;35.8)	37.3 (34.8;39.8)	33.1 (29.5;36.7)
**Musculoskeletal**	56.3 (54.9;57.7)	63.3 (60.7;65.9)	50.6 (46.2;55.0)
**Neurological**	21.4 (20.3;22.5)	32.0 (29.6;34.4)	40.2 (36.0;44.4)
**Psychiatric**	19.8 (18.8;20.8)	20.5 (18.4;22.6)	22.0 (18.2;25.9)
**Respiratory**	18.5 (17.4;19.5)	20.5 (18.4;22.6)	22.4 (18.6;26.2)
**Sensorial**	82.8 (81.7;83.9)	85.2 (83.3;87.2)	82.6 (79.0;86.3)
**Sequelae of injury**	9.7 (8.9;10.5)	10.7 (8.9;12.5)	10.2 (6.9;13.5)
**Urological**	12.4 (11.5;13.3)	21.4 (19.1;23.7)	30.0 (25.8;34.2)

95% CI = 95% confidence interval;

a Self-reported disability = positive response to the question “Do you consider that you have a disability?”;

b Disability = any restriction in doing at least one activity of daily living (ADL) with or without technical aid;

c Severe disability = inability to perform at least one ADL alone.

d Prevalence of situations of disability in the overall adult population living in households in France.

### Contribution of chronic conditions to disability

The highest AFs were for sensorial impairments (49.7% (49.5;50.0) to 57.3% (57.2;57.5)) (Table S2). Even though the prevalence of neurological (10.1%) or psychiatric diseases (6.3%) was not high, the AF findings for these diseases were significant because of the high relative risk of being disabled with these diseases. In contrast, respiratory disorders had a higher prevalence (11.5%) but lower AFs (<15.0%). We grouped conditions with AFs≤15.0% in each age group: cancer and digestive, respiratory and dermatological diseases.

**Figure 2:**
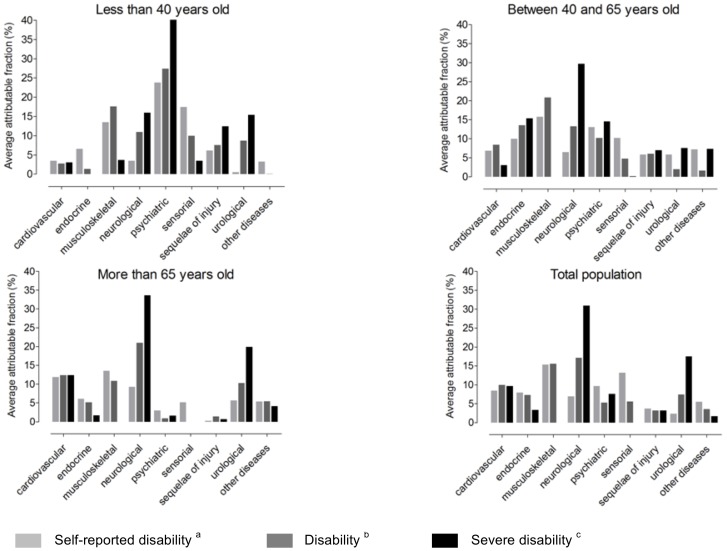
Average attributable fractions of chronic conditions for different categories of disability in France and according to age. ^a^ Self-reported disability = positive response to the question “Do you consider that you have a disability?”; ^b^ Disability = any restriction in doing at least one activity of daily living (ADL) with or without technical aid (self-report assessment); ^c^ Severe disability = inability to perform at least one ADL alone (self-report assessment).

AAFs estimates for disability, severe disability and self-reported disability are in table 4, 5 and 6, respectively, and AAFs are represented in Figure 2.

**Table 4:** Average attributable fraction^a^ (AAF) estimates (%) for each chronic disorder for disability^b^ in France.

Age class	≤40	40–65	>65	Total Population
Rank	(*N = 143 859*)^d^	(*N = 534 675*)^d^	(*N = 1 158 219*)^d^	*(N = 1 836 753)* d
**1**	**Psychiatric**	**Musculoskeletal**	**Neurological**	**Neurological**
	**27.4**	**20.9**	**21.0**	**17.4**
**2**	**Musculoskeletal**	**Endocrine**	**Cardiovascular**	**Musculoskeletal**
	**17.6**	**13.6**	**12.4**	**16.4**
**3**	**Neurological**	**Neurological**	**Musculoskeletal**	**Cardiovascular**
	**11.0**	**13.3**	**10.9**	**11.1**
**4**	Sensorial	Psychiatric	Urological	Endocrine
	10.0	10.2	10.3	8.2
**5**	Urological	Cardiovascular	Other diseases^c^	Urological
	8.7	7.1	5.5	7.4
**6**	Sequelae of injury	Sequelae of injury	Endocrine	Psychiatric
	7.6	6.1	5.2	5.8
**7**	Cardiovascular	Sensorial	Sequelae of injury	Sensorial
	2.8	4.8	1.4	3.8
**8**	Endocrine	Urological	Psychiatric	Sequelae of injury
	1.4	2.0	0.9	3.1
**9**	Other diseases^c^	Other diseases^c^	Sensorial	Other diseases^c^
	0.1	1.7	0.0	3.3
**Sum**	**86.6**	**79.7**	**67.6**	**75.3**

a AAFs were computed as described by Rückinger [8] for SAS software that we modified to take into account sample design and adjusting variables. AAFs were adjusted for sex, place of residence, marital status, living situation and educational level. We also adjusted on age (by class) for the total population;

b Disability = any restriction for doing at least one activity of daily living (ADL), with or without technical aid;

c Other diseases = respiratory, dermatological, digestive disorders and cancer;

d *N* = the estimated number of people in the French adult population living in household. Numbers reflect the use of a non-rounded population in calculations.

**Table 5:** Average attributable fraction^a^ (AAF) estimates (%) for each chronic disorder for severe disability^b^ in France.

Age class	≤40	40–65	>65	Total Population
Rank	(*N = 67 681*)^d^	(*N = 112 254*)^d^	(*N = 493 264*)^d^	(*N = 673 199*)^d^
**1**	**Psychiatric**	**Neurological**	**Neurological**	**Neurological**
	**40.3**	**29.7**	**33.6**	**31.0**
**2**	**Neurological**	**Endocrine**	**Urological**	**Urological**
	**16.0**	**15.4**	**19.9**	**17.5**
**3**	**Urological**	**Psychiatric**	**Cardiovascular**	**Cardiovsacular**
	**15.4**	**14.6**	**12.4**	**9.7**
**4**	Sequelae of injury	Urological	Other diseases^c^	Psychiatric
	12.5	7.6	4.2	7.6
**5**	Musculoskeletal	Other diseases^c^	Endocrine	Endocrine
	3.7	7.4	1.7	3.4
**6**	Sensorial	Sequelae of injury	Psychiatric	Sequelae of injury
	3.5	7.0	1.6	3.2
**7**	Cardiovascular	Cardiovascular	Sequelae of injury	Other diseases^c^
	3.1	3.1	0.7	1.7
**8**	Endocrine	Sensorial	Musculoskeletal	Musculoskeletal
	0.0	0.2	0.0	0.0
**9**	Other diseases^c^	Musculoskeletal	Sensorial	Sensorial
	0.0	0.0	0.0	0.0
**Sum**	**94.5**	**85.0**	**74.1**	**74.1**

a AAFs were computed as described by Rückinger [8] for SAS software that we modified to take into account sample design and adjusting variables. AAFs were adjusted for sex, place of residence, marital status, living situation and educational level. We also adjusted on age (by class) for the total population;

b Severe disability = inability to perform at least one ADL alone;

c Other diseases = respiratory, dermatological, digestive disorders and cancer;

d *N* = the estimated number of people in the French adult population living in household. Numbers reflect the use of a non-rounded population in calculations.

**Table 6:** Average attributable fraction^a^ (AAF) estimates (%) of chronic conditions for self-reported disability^b^ in France.

Age class	≤40	40–65	>65	Total Population
Rank	(*N = 804 615*)^d^	(*N = 2 440 944*)^d^	(*N = 2 473 309*)^d^	(*N = 5 718 868)* d
**1**	**Psychiatric**	**Musculoskeletal**	**Musculoskeletal**	**Musculoskeletal**
	**23.8**	**15.8**	**13.6**	**15.4**
**2**	**Sensorial**	**Psychiatric**	**Cardiovascular**	**Sensorial**
	**17.5**	**13.1**	**11.9**	**13.2**
**3**	**Musculoskeletal**	**Sensorial**	**Neurological**	**Psychiatric**
	**13.5**	**10.2**	**9.3**	**9.7**
**4**	Endocrine	Endocrine	Endocrine	Cardiovascular
	6.6	10.0	6.1	8.5
**5**	Sequelae of injury	Other diseases^c^	Urological	Endocrine
	6.2	7.2	5.7	8.0
**6**	Cardiovascular	Cardiovascular	Other diseases^c^	Neurological
	3.5	6.9	5.4	7.0
**7**	Neurological	Neurological	Sensorial	Other diseases^c^
	3.5	6.5	5.2	5.5
**8**	Other diseases^c^	Sequelae of injury	Psychiatric	Sequelae of injury
	3.3	5.9	3.0	3.7
**9**	Urological	Urological	Sequelae of injury	Urological
	0.5	5.9	0.3	2.4
**Sum**	**78.4**	**81.5**	**60.5**	**73.4**

a AAFs were computed as described by Rückinger [8] for SAS software that we modified to take into account sample design and adjusting variables. AAFs were adjusted for sex, place of residence, marital status, living situation and educational level. We also adjusted on age (by class) for the total population;

b Self-reported disability = a positive answer to the question “Do you consider to have a disability?”;

c Other diseases = respiratory, dermatological, digestive disorders and cancer;

d *N* = the estimated number of people in the French adult population living in household. Numbers reflect the use of a non-rounded population in calculations.

Neurological, musculoskeletal, and cardiovascular diseases had the greatest impact on disability (AAF 17.4%, 320 000 people; 16.4%, 290 000 people; and 11.1%, 180 000 people). The AAF for neurological conditions was high for people >65 years old (21.0%, 240 000 people), whereas that for psychiatric disorders was highest for people ≤40 years old (27.4%, 40 000 people).

Neurological diseases had the largest impact on severe disability (AAF 31.0%, 210 000 people), except for people ≤40 years old, for whom psychiatric disorders had the most impact (40.3%, 30 000 people). The burden of musculoskeletal disorders to severe disability was insignificant.

Musculoskeletal conditions had the largest impact on self-reported disability (AAF 15.4%, 880 000 people), then sensorial and psychiatric diseases (13.2%, 750 000 people; and 9.7%, 550 000 people).

Our study revealed quantitative and qualitative differences between AAF and AF findings. Quantitatively, AAFs were lower than AFs for all chronic diseases, particularly for sensorial disorders (3.8% vs. 57.3, respectively, for disability in the total population), musculoskeletal disorders (16.4% vs. 48.5%), and cardiovascular disorders (11.1% vs. 40.6%). In contrast, AAFs and AFs for neurological diseases were close (17.4% vs. 24.4%, respectively, for disability in the total population). The sum of AAFs, which represent the part of disability attributable to chronic conditions, ranged from 67.6% to 94.5% and that of AFs from 117.6% to 257.9%. Qualitatively, AAFs and AFs did not differ for severe disability, whatever the age class (that is the rank of chronic disorders AAFs and AFs was the same); they were mostly similar for chronic disorders. AFs were higher than AAFs for disability and self-reported disability for sensorial disorders but not other chronic diseases.

## Discussion

Although several studies suggest that the prevalence of disability is decreasing in developed countries [11], our findings are not so optimistic. In 1999, 3.7% of people reported at least one ADL restriction in France [21], but 3.9% reported such restrictions in 2008–2009. The disability rate we found among people >65 years old in France (12.5%) is between that for Finland (10.2%) and England (15.3%) [11]. The prevalence of severe disability among elderly people in France (5.3%) is comparable to that in Canada (5.8%) [11].

We used three indicators to identify the population reporting disability [22] – restriction in ADL and impossibility to perform ADL without help, as well as self-reported disability – which are pivotal in the International Classification of Functioning, Disability and Health (ICF) [23] and probably reflect other domains [1]. We found that the chronic conditions neurological, musculoskeletal, cardiovascular and psychiatric disorders had the largest contribution to disability in France in 2008–2009. Together, they contributed to 50.7% of disability, 48.3% of severe disability and 40.6% of self-reported disability (63.9%, 65.2% and 55.3%, respectively, of the part attributable to chronic conditions). These rates reflect the expected proportion of disability that could be reduced in the ideal situation where chronic disorders could be totally eliminated in France. Although this target is not realistic, a first step could be to improve prevention and treatment of these health conditions that lead to disability.

Our results can only be grossly compared with previous results from developed countries because our approach differs methodologically from prior efforts. In a 2006 Canadian study, the most common disability-related health conditions were arthritis, back problems and hearing disorders [6], whereas in a 2001 Organization for Economic Co-operation and Development study, rheumatism was the leading cause of disability-associated conditions in elderly people in the United States [11]. In our study, the low impact of sensorial disorders and heavy burden of neurological diseases contrast with these results. These differences might be due to the lack of adjusted analyses in these studies. In fact, the high contribution of sensorial disorders in our gross analysis of disability was greatly eliminated after adjusting for age and co-morbidities, which suggests that these factors are confounders.

The respective contribution of chronic conditions differs by age, as well as definition and level of disability. Although musculoskeletal diseases had the heaviest burden in self-reported disability and the second highest impact on disability, its contribution to severe disability was almost nil. This finding suggests that people with musculoskeletal impairments often have restrictions they consider disabling (e.g., because of pain) but are rarely completely dependent in ADL. In contrast, the important contribution of neurologic conditions to severe disability in elderly people may be explained by the high level of dependence of people with neurodegenerative disorders. The high contribution of sensorial disorders to self-reported disability contrasts with the low impact on ADL restriction (severe or not). This finding shows the limitations of the Katz ADL score in that it does not include activities such as reading, sewing, cooking, or using the telephone, which involve sensorial functions. Thus, the impact of those disorders might be underestimated. We show the same findings for psychiatric disorders, which first disturb social activities that are not considered in the Katz ADL score. Finally, we highlight the significant impact of psychiatric impairments in people ≤40 years old, as was suggested by recent data [2], [24], [25], which emphasizes the need for efforts to reduce the burden of mental health problems in developed countries.

Our results cannot suggest a single priority for public health priorities in terms of which chronic condition to eliminate first. However, policymakers need information on the implications of each disability definition and the level of severity that could be relevant in disability-related programs. From our results, policymakers should focus on neurological disorders among elderly people to reduce dependence; should prevent psychiatric disorders among young people to reduce disability in this age class; and should act on musculoskeletal disorders in the overall population to improve the feeling of wellbeing, which may also reduce work absenteism in the working-age population. Examining these findings in terms of economic data would be of interest.

The main strength of this study is that our results are representative of the French household population. Thus, our data are valuable to policymakers and ensure comparison with data from other countries. Another advantage is our use of the AAF for analysis, which provides a framework for considering co-morbid situations [7]. Most studies do not consider co-morbidities [5], or they use approaches not strictly valid in this situation, such as gross AF or adjusted odds ratios [4]. However, these methods can overestimate the potential impact of preventive strategies [8]. In fact, we found sums of unadjusted AFs higher than sums of AAFs and always exceeded 100%. Such a result is not realistic and highlights the interest of AAF in analyzing co-morbidity data. On the contrary, sums of AAFs for chronic disorders never reached 100%, which suggests that other factors, such as socioeconomic, environmental, and personal factors, have an impact on disability. The contribution of these factors was about 25% for the overall population and increased with age.

Our study contains some limitations. First, the Disability-Health Survey questionnaire did not collect data on several chronic disorders such as inflammatory bowel diseases or end stage chronic renal failure. However, the prevalence of these disorders is low in France [26], [27], so they probably contribute little to disability and their inclusion might not have changed our results greatly. We grouped diseases with AFs≤15.0% assuming that their contribution to disability would be insignificant as compared with other conditions, which was confirmed. Because AAFs for respiratory diseases were close to 15.0%, we calculated the independent AAFs for these diseases (data not shown) and found them to be <2%, and the AAFs for other chronic disorders did not change. Finally, we used self-reports of chronic disorders and disability. According to previous data, the accuracy of self-reporting is high for chronic disorders such as stroke [28], coronary heart disease [29] or cancer [28] but low for conditions such as arthritis [28] or non-coronary heart diseases [29]. The reliability of self-reporting in our survey is unknown. However, the prevalence rates for diseases were similar to those from other French [30] and European surveys [2]. Moreover, we used a checklist of diseases, which has been shown to yield more complete and accurate reports than estimates derived from responses to open-ended questions [31], and only diagnoses of chronic conditions by physicians were retained.

## Conclusions

Neurological, musculoskeletal, cardiovascular and psychiatric disorders are the main chronic disorders contributing to disability in France. Neurological diseases have the largest impact on severe disability and in elderly people, whereas psychiatric impairments have a heavy burden in people ≤40 years old. Although disability has been decreasing in developed countries, our results show that a substantial proportion of the French population feels disabled and is restricted in ADL. These findings should help health policymakers decide on priorities for health-service delivery in France and in other developed countries. These data emphasize the need to support international and national efforts to better address the main challenges with chronic diseases and disability.

## Supporting Information

Appendix S1 Method of calculation of the average attributable fraction (AAF)
(DOC)

Table S1 Sociodemographic characteristics of the population in France for each chronic condition
(DOC)

Table S2 Gross attributable fractions (AFs) for each chronic condition estimated for the different disability categories
(DOC)
