# Supplementary material for: Respective Contribution of Chronic Conditions to Disability in France: Results from the National Disability-Health Survey
Source: PLoS One. 2012 Sep 14;7(9):e44994. doi: 10.1371/journal.pone.0044994 (PMC3443206; doi:10.1371/journal.pone.0044994)
Supplement: Appendix S1 — Method of calculation of the average attributable fraction (AAF) (DOC) [file pone.0044994.s001.doc]

**Appendix S1.** Method of calculation of the average attributable fraction (AAF)

We described the statistical development of the AAF as it was developed in Eide’s paper [1] using an example inspired by DH survey. It includes 3 steps of calculation: 1) the adjusted attributable fraction; 2) the sequential attributable fraction; 3) the average attributable fraction.

Suppose the explanatory variables could be divided into a set of L exposures of interest (E_k_). We took as example a situation where disability (D) could be explained by 2 exposures: musculoskeletal disorders (M) and cardiovascular diseases (C).

**1. Adjusted attributable fraction.**

We first calculated the adjusted attributable fraction (λ) that could be interpreted as the proportion of the disabled population that would be prevented if the risk of being disabled in the exposed population (that is people with the disease of interest) were changed to the risk of the unexposed population (that is people that do not have the disease of interest).

A useful reformulation is [2]:

may be named "the component of λ due to the class E_k_".

There are 2^k^-1 possible associations of exposures.

For example, for people with musculoskeletal disorders (M) and/or cardiovascular diseases (C), there were k=2^2^ -1=3 possible associations: MC_0_, M_0_C, MC

So we could write:

Where:

**2. Sequential attributable fraction**

The next step was to calculate the sequential attributable fraction. We were interesting in the reduction of cases when preventing all the exposures, one at a time, in a given sequence. The difference λ(*^l'^*)- λ(*^l^*) *(l<l'*) corresponds to the additional effect of removing the...*l'*-th exposures in the population after having removed the first *l* exposures in a specified sequence.

These differences might be called sequential attributable fractions (safs). The saf would differ for the same set of exposures according to which exposures are removed first. Among the L exposure variables there would be L! different removal orderings of the exposures.

In our example, there were 2! =2 different removal orderings: either M or C could be removed first.

Removing M first we obtained:

Whereas removing C first we obtained:

**3. Average attributable fraction**

A way to take into account the different safs for the same exposure over all possible removal orderings is to average these safs: this is the average attributable fraction (AAF) [1,3]. The justification to use AAF was developed by Cox [4] for mathematical game theory but can be easily transferred to epidemiological context.

In our example:

**References**

1. Eide GE, Gefeller O (1995) Sequential and average attributable fractions as aids in the selection of preventive strategies. J Clin Epidemiol 48: 645-655.

2. Miettinen OS (1974) Proportion of disease caused or prevented by a given exposure, trait or intervention. Am J Epidemiol 99: 325-332.

3. Gefeller O, Land M, Eide GE (1998) Averaging attributable fractions in the multifactorial situation: assumptions and interpretation. J Clin Epidemiol 51: 437-441.

4. Cox J (1985) A new measure of attributable risk for public health application. Manage Sci 31: 800-813.
