# Supplementary material for: Respective Contribution of Chronic Conditions to Disability in France: Results from the National Disability-Health Survey
Source: PLoS One. 2012 Sep 14;7(9):e44994. doi: 10.1371/journal.pone.0044994 (PMC3443206; doi:10.1371/journal.pone.0044994)
Supplement: Table S1 — Sociodemographic characteristics of the population in France for each chronic condition (DOC) [file pone.0044994.s002.doc]

**Table S1.** Socio-demographic characteristics of the population in France for each chronic condition

… /…

|  |  | **Cancer** | **Cardiovascular** | **Dermatological** | **Digestive** | **Endocrine** | **Musculoskeletal** | **Neurological** |
| --- | --- | --- | --- | --- | --- | --- | --- | --- |
| **Gender (%)** | Men | 47.6 (42;53.1) | 42.0 (40.3;43.8) | 40.7 (36.8;44.5) | 41.6 (37.7;45.5) | 41.0 (39.0;43) | 38.8 (37.2;40.4) | 33.1 (30.5;35.8) |
| **Age (mean), years** |  | 63.3 (61.6;64.9) | 63.3 (62.8;69.9) | 45.9 (44.7;47.2) | 54.4 (53;67.0) | 56.1(55.5;56.7.0) | 57.5 (57;58.0) | 49.0 (48.1;49.9) |
| **In couple (%)** |  | 69.2 (64.3;74.1) | 67.2 (65.5;68.8) | 67.2 (63.6;70.8) | 70.4 (67.2;73.6) | 68.0 (66.2;69.9) | 66.7 (65.2;68.2) | 64.7 (62.0;67.3) |
| **Live alone (%)** |  | 4.7 (2.3;7.2) | 6.9 (6.0;7.8) | 6.4 (4.6;8.3) | 7.2 (5.2;9.2) | 7.1 (6.0;8.2) | 6.5 (5.7;7.3) | 7.5 (6.0;9.0) |
| **Educational attainment (%)** | Primary school or less | 48.2 (42.9;53.5) | 48.0 (46.3;49.8) | 24.0 (21.3;26.8) | 42.9 (39.2;46.5) | 44.9 (43.0;46.8) | 40.9 (39.4;42.4) | 35.2 (32.9;37.6) |
|  | Middle/high school | 36.6 (31.4;41.8) | 38.5 (36.8;40.3) | 44.7 (40.8;48.5) | 38.9 (35.0;42.8) | 40.6 (38.2;42.6) | 42.1 (40.5;43.7) | 42.5 (39.7;45.2) |
|  | Higher education | 15.2 (10.8;19.6) | 13.4 (12.1;14.8) | 31.3 (27.5;35.1) | 18.2 (14.8;21.7) | 14.5 (12.9;16.1) | 17.0 (15.6;18.3) | 22.3 (19.7;24.9) |
| **Working status (%)** | Unemployed | 4.3 (2.1;6.5) | 3.6 (2.9;4.3) | 6.8 (4.8;8.7) | 5.7 (3.7;7.7) | 5.8 (4.8;6.8) | 4.5 (3.9;5.2) | 7.0 (5.6;8.5) |
|  | Retired | 58.8 (53.5;64.0) | 57.0 (55.3;58.8) | 23.0 (20.2;25.8) | 36.2 (32.9;39.5) | 40.7 (38.8;42.5) | 42.1 (40.6;43.6) | 24.9 (23.0;26.8) |
|  | Housewife | 8.3 (5.0;11.6) | 7.2 (6.3;8.1) | 6.87 (5.1;8.7) | 7.81 (6.0;9.6) | 9.0 (7.8;10.1) | 6.8 (6.1;7.5) | 7.9 (6.4;9.3) |
|  | Employed/student | 28.7 (23.8;33.5) | 32.2 (30.4;33.9) | 63.4 (59.9;66.8) | 50.3 (46.6;54.0) | 44.6 (42.6;46.6) | 46.5 (45.0;48.1) | 60.2 (57.8;62.7) |
| **Place of residence (%)** | Rural area | 25.4 (20.9;30.0) | 25.5 (24.1;27.0) | 24.0 (21.0;27.1) | 24.5(21.4;27.6) | 26.3(24.6;28.0) | 26.1(24.7;27.4) | 23.0 (20.8;25.1) |
|  | Small urban area | 38.0 (32.6;43.4) | 41.9 (40.1;43.6) | 37.4 (33.8;41.0) | 38.5(34.7;42.3) | 39.5(37.6;41.4) | 39.0(37.5;40.5) | 40.0 (37.3;42.6) |
|  | Big urban area | 36.6 (31.5;41.6) | 32.6 (30.9;34.3) | 38.6 (34.8;42.3) | 37.0 (33.1;40.8) | 34.2(32.3;36.1) | 34.9(33.4;36.4) | 37.1 (34.3;39.8) |

Data in brackets are 95% confidence intervals.

**Table S1 (continued).** Socio-demographic characteristics of the population in France for each chronic condition

|  |  | **Psychiatric** | **Respiratory** | **Sensorial** | **Sequelae of injury** | **Urological** | **At least 1 chronic condition** | **Total population** |
| --- | --- | --- | --- | --- | --- | --- | --- | --- |
| **Gender (%)** | Men | 33.5 (30.6;36.5) | 43.1 (40.4;45.9) | 44.1 (42.9;45.3) | 59.5 (55.2;63.7) | 36.6 (33.0;40.2) | **45.3 (44.2;46.3)** | **47.5 (46.5;48.5)** |
| **Age (mean), years** |  | 54.6 (53.6;55.6) | 49.5 (48.6;50.4) | 53.7 (53.3;54.1) | 51.7 (50.1;53.3) | 60.5 (59.0;62.0) | **51.4 (51.0;51.8))** | **48.4 (48.1;48.7)** |
| **In couple (%)** |  | 55.1 (52.1;58.1) | 66.0 (63.4;68.6) | 68.3 (67.2;69.4) | 66.5 (62.3;70.7) | 64.6 (60.9;68.2) | **67.9 (66.9;68.9)** | **66.7 (65.8;67.7)** |
| **Live alone (%)** |  | 6.4 (4.9;8.0) | 6.5 (5.1;7.8) | 7.3 (6.7;8.0) | 7.9 (5.3;10.5) | 6.2 (4.2;8.2) | **7.4 (6.9;8.0)** | **7.3 (6.8;7.9)** |
| **Educational attainment (%)** | Primary school or less | 44.4 (41.6;47.2) | 30.0 (27.8;32.2) | 32.7 (31.7;33.7) | 33.3 (29.4;37.2) | 45.5 (42.1;48.3) | **31.3 (30.5;32.2)** | **28.6 (27.8;29.4)** |
|  | Middle/high school | 39.1 (36.2;42.1) | 43.1 (40.4;45.9) | 43.4 (42.2;44.5) | 49.4 (44.9;53.9) | 36.0 (32.2;39.7) | **44.9 (43.8;45.9)** | **46.6 (45.7;47.6)** |
|  | Higher education | 16.5 (13.8;19.1) | 26.9 (24.2;29.5) | 23.9 (22.8;25.0) | 17.2 (13.6;20.9) | 18.6 (15.1;22.1) | **23.8 (22.8;24.8)** | **24.8 (23.8;25.7)** |
| **Working status (%)** | Unemployed | 6.9 (5.4;8.3) | 4.8 (3.6;6.0) | 4.5 (4.0;5.0) | 6.4 (4.5;8.4) | 3.9 (2.1;5.7) | **5.0 (4.5;5.5)** | **5.6 (5.1;6.0)** |
|  | Retired | 34.2 (31.5;36.9) | 28.9 (26.9;30.9) | 35.0 (33.9;36.0) | 30.8 (27.2;34.4) | 54.2 (50.6;57.8) | **31.1 (30.2;32.0)** | **26.2 (25.5;27.0)** |
|  | Housewife | 8.3 (6.7;9.9) | 7.3 (6.0;8.7) | 6.1 (5.6;6.7) | 3.1 (1.8;4.4) | 6.7 (4.9;8.5) | **6.3 (5.8;6.8)** | **6.2( 5.7;6.6)** |
|  | Employed/student | 50.6 (47.7;53.6) | 59.0 (56.5;61.4) | 54.4 (53.3;55.5) | 59.7 (55.7;63.6) | 35.2 (31.4;38.9) | **57.6 (56.6;58.6)** | **62.0 (61.1;62.9)** |
| **Place of residence (%)** | Rural area | 25.5 (23.0;28.0) | 25.0 (22.8;27.3) | 23.8 (22.3;25.3) | 30.4 (26.4;34.4) | 26.7 (23.6;29.9) | **25.6 (24.7;26.5)** | **24.9 (24.1;25.7)** |
|  | Small urban area | 38.9 (35.9;41.8) | 34.4 (32.0;36.9) | 36.4 (34.7;38.1) | 36.4 (32.2;40.6) | 38.6 (35.0;42.3) | **37.9 (36.9;38.8)** | **37.5 (36.6;38.4)** |
|  | Big urban area | 35.6 (32.8;38.5) | 40.5 (37.8;43.2) | 39.8 (38.0;41.5) | 33.2 (28.9;37.5) | 34.6 (30.9;38.3) | **36.5 (35.5;37.6)** | **37.6 (36.6;38.5)** |
