# Supplementary material for: Respective Contribution of Chronic Conditions to Disability in France: Results from the National Disability-Health Survey
Source: PLoS One. 2012 Sep 14;7(9):e44994. doi: 10.1371/journal.pone.0044994 (PMC3443206; doi:10.1371/journal.pone.0044994)
Supplement: Table S2 — Gross attributable fractions (AFs) for each chronic condition estimated for the different disability categories (DOC) [file pone.0044994.s003.doc]

Table S2. Gross attributable fractions^a^ (AFs) for each chronic condition estimated for disability^b^ and severe disability^c^ in France

|  | **Disability^b^ % (95% CI)** | | | | **Severe disability^c^ % (95% CI)** | | | |
| --- | --- | --- | --- | --- | --- | --- | --- | --- |
| **Age class**  **(years)** | **≤40** | **40-65** | **>65** | **Total**  **population** | **≤40** | **40-65** | **>65** | **Total**  **population** |
|  | (***N=****143 859)*^d^ | *(N=534 675)*^d^ | *(N= 1 158 21*) ^d^ | *(N=1 836 753)* ^d^ | *(N=67 681)*^d^ | *(N=112 254)*^d^ | *(N=493 26*)^d^ | (*N=673 199)*^d^ |
| **Musculoskeletal** | 40.6(40.3;40.9) | 44.4(44.2;44.6) | 30.6(30.6;30.8) | 48.5(48.4;48.6) | 22.4(22.0;22.8) | 14.8(14.4;15.2) | 9.3(9.0;9.6) | 30.7(30.5;30.8) |
| **Sensorial** | 25.5(25.1;26.0) | 28.0(27.6;28.5) | 17.1(16.7;17.5) | 57.3(57.2;57.5) | 17.9(17.2;18.5) | 11.8(10.7;12.8) | 0.4(0.0;1.1) | 49.7(49.5;50.0) |
| **Neurological** | 31.9(31.6;32.1) | 24.5(24.4;24.6) | 23.0(23.0;23.1) | 24.4(24.3;24.4) | 40.4(40.0;40.8) | 36.9(36.6;37.2) | 31.5(31.3;31.6) | 33.4(33.3;33.6) |
| **Cardiovascular** | 14.0(13.8;14.2) | 21.2(21.2;21.3) | 26.9(26.8;27.1) | 40.6(40.5;40.7) | 12.6(12.3;12.9) | 15.5(15.2;15.9) | 23.5(23.2;23.7) | 40.6(40.5;40.8) |
| **Psychiatric** | 35.0(34.8;35.3) | 20.4(20.3;20.5) | 7.4(7.3;7.5) | 15.1(15.1;15.2) | 46.0(45.6;46.4) | 24.4(24.1;24.7) | 8.5(8.4;8.6) | 16.8(16.7;16.9) |
| **Urological** | 15.7(15.5;15.9) | 7.0(7.0;7.1) | 17.2(17.1;17.3) | 17.4(17.4;17.5) | 23.8(23.5;24.1) | 14.0(13.7;14.2) | 24.6(245;24.8) | 26.5(26.3;26.6) |
| **Endocrine** | 6.0(5.8;6.2) | 26.5(26.2;26.5) | 11.1(11.0;11.2) | 22.8 (22.7;22.9) | 0.0(0.0;0.0) | 25.2(24.8;25.6) | 6.4(6.2;6.6) | 17.6(17.4;17.7) |
| **Sequelae of injury** | 17.0(16.8;17.2) | 10.8(10.7;10.9) | 3.7(3.6;3.7) | 7.4(7.4;7.5) | 22.4(22.0;22.7) | 13.1(12.9;13.3) | 2.4(2.3;2.5) | 6.9(6.8;7.0) |
| ***Respiratory*** | *7.4(7.2;7.7)* | *8.7(8.6;8.8)* | *8.9(8.8;9.0)* | *10.2(10.1;10.3)* | *2.9(2.6;3.2)* | *9.9(9.7;10.2)* | *11.7(11.6;11.8)* | *12.4(12.2;12.5)* |
| ***Digestive*** | *6.9(6.7;7.0)* | *7.2(7.1;7.3)* | *5.0(5.0;5.1)* | *7.2(7.1;7.2)* | *3.5(3.4;3.7)* | *6.3(6.1;6.5)* | *4.2(4.1;4.3)* | *6.1(6.0;6.1)* |
| ***Dermatological*** | *4.7(4.5;4.9)* | *5.9(5.8;5.9)* | *3.8(3.8;3.9)* | *3.7(3.7;3.8)* | *4.0(3.7;4.2)* | *7.2(7.0;7.4)* | *1.8(1.8;1.9)* | *2.1(2.1;2.2)* |
| ***Cancer*** | *2.0(1.9;2.1)* | *2.5(2.5;2.6)* | *1.2(1.2;1.3)* | *3.3(3.3;3.4)* | *3.1(3.0;3.3)* | *2.1(2.0;2.2)* | *1.5(1.4;1.6)* | *3.6(3.6;3.7)* |
| **Sum** | **206.7** | **207.1** | **155.9** | **257.9** | **199.0** | **181.2** | **125.8** | **246.4** |

95% CI= 95% confidence interval

^a^ AFs were calculated by use of the Levin formula^5^: where Pe is the prevalence of the disease in the overall population and RR the relative risk of being disabled with the disease. Confidence intervals of AF were calculated with use of the Fleiss formula^6^

^b^ Disability= any restriction for doing at least one activity of daily living (ADL), with or without technical aid.

^c^ Severe disability=inability to perform at least one ADL alone

^d^ *N*=the estimated number of people in the French adult population living in household. Numbers reflect the use of a non-rounded population in calculations.

^e^ Self-reported disability= a positive answer to the question "Do you consider that you have a disability?"

Diseases with AF<15% in each age group are in italics. They were grouped for AAF calculation under “Other diseases.”

**Table S2. (continued)** Gross attributable fraction^a^ (AF) for each chronic condition estimates for self-reported disability^e^ in France

|  | **Self-reported disability**^e^ **% (95% CI)** | | | |
| --- | --- | --- | --- | --- |
|  | **≤40** | **40-65** | **>65** | **Total**  **Population** |
|  | *(N=804 615*)^d^ | *(N=2 440 94)^d^* | (*N= 2 473 309)^d^* | (*N=5 718 868)^d^* |
| **Musculoskeletal** | 25.4(25.3;25.5) | 34.8(34.7;34.9) | 26.3(26.2;26.4) | 38.7(38.6;38.7) |
| **Sensorial** | 27.7(27.5;27.9) | 30.3(30.1;30.5) | 18.6(18.3;18.8) | 50.3(50.2;50.4) |
| **Neurological** | 14.0(14.0;14.1) | 14.2(14.1;14.2) | 10.1(10.1;10.2) | 12.6(12.6;12.6) |
| **Cardiovascular** | 8.8(8.7;8.8) | 17.4(17.3;17.5) | 21.7(21.6;21.8) | 28.6(28.6;28.7) |
| **Psychiatric** | 20.8(20.7;20.9) | 17.6(17.5;17.6) | 6.3(6.3;6.4) | 14.4(14.4;14.5) |
| **Urological** | 2.5(2.4;2.5) | 3.8(3.8;3.8) | 9.2(9.1;9.2) | 7.9(7.9;8.0) |
| **Endocrine** | 11.0(10.9;11.1) | 19.1(19.0;19.1) | 10.1(10.1;10.2) | 19.3(19.2;19.3) |
| **Sequelae of injury** | 10.1(10.0;10.1) | 8.3(8.3;8.4) | 2.3(2.3;2.3) | 6.4(6.4;6.4) |
| ***Respiratory*** | *7.4(7.3;7.5)* | *8.2(8.1;8.2)* | *6.0(6.0;6.1)* | *7.9(7.9;7.9)* |
| ***Digestive*** | *4.8(4.7;4.9)* | *7.2(7.2;7.2)* | *3.4(3.4;3.4)* | *6.1(6.1;6.2)* |
| ***Dermatological*** | *2.6(2.6;2.7)* | *4.7(4.7;4.8)* | *2.0(2.0;2.0)* | *2.7(2.7;2.7)* |
| ***Cancer*** | *1.1(1.1;1.1)* | *2.7(2.7;2.7)* | *1.6(1.6;1.7)* | *3.0(3.0;3.1)* |
| **Sum** | **136.2** | **168.3** | **117.6** | **197.9** |
